# Leveraging Multimodal Large Language Models for Fall Risk Reduction in Older Adults in the Home: Proposed Model Design

**DOI:** 10.2196/77591

**Published:** 2026-05-13

**Authors:** Justin Do, Vivaswat Suresh, Lily Zhang, Bharvi M Chavre, Jeremy Cha, Robert Pugliese

**Affiliations:** 1 Sidney Kimmel Medical College, Thomas Jefferson University, 925 Chestnut St., Basement Vault, Philadelphia, PA, 19107, United States, 1 4084718003, 1 2154841949; 2 Health Design Lab, Thomas Jefferson University, Philadelphia, PA, United States

**Keywords:** LLM, multimodal, image generation, fall risk, older adults, large language model

## Abstract

This research letter proposes a novel model design leveraging natively multimodal large language models to identify fall risks and generate visualizations of recommended home environmental modifications, aiming to improve the accessibility and impact of personalized fall prevention advice for older adults. Through a pilot rating study, this work demonstrates that multimodal large language models can generate safe and actionable advice to reduce fall risk in lived spaces of older adults, and also generate realistic edits based on original images. While this concept needs further testing and clinical comparison, it highlights a promising avenue for further innovation of fall prevention tactics.

## Introduction

Falls among older adults cause significant mortality and increased health care costs [1]. Current literature has identified combined behavioral and exercise interventions as effective preventions for fall risks, improving balance performance, and reducing fear of falls [2], while limited evidence exists regarding medication-induced falls [3]. Home environmental intervention is also effective: safety assessments have been shown to reduce fall rate by 23%‐36% [1], with applied home modifications contributing a 7% risk reduction [4]. However, both external (insurance) and self-imposed (ie, the perception that safety assessments are invasive) barriers impede widespread implementation [5]. While research on frailty assessments is robust, gaps remain in technology-enabled interventions [6]. Prior studies have shown acceptability by older adults to embrace digital and electronic tools [5]. Existing remote home assessment protocols rely on caregiver camera operation, written instructions comprehension, and professional review of footage [7], while telehealth occupational therapy (OT) assessments may require insurance authorization, creating both obstacles and delays. Multimodal large language models (LLM) can fuse visual and text information, offering a scalable alternative while preventing encroachment on user values. This study aims to evaluate the ability of LLMs to produce safe, clinically useful, and actionable outputs that identify fall risks from user-provided home imagery and uniquely generate visualizations of the recommended environmental modifications.

## Methods

**Figure 1.**
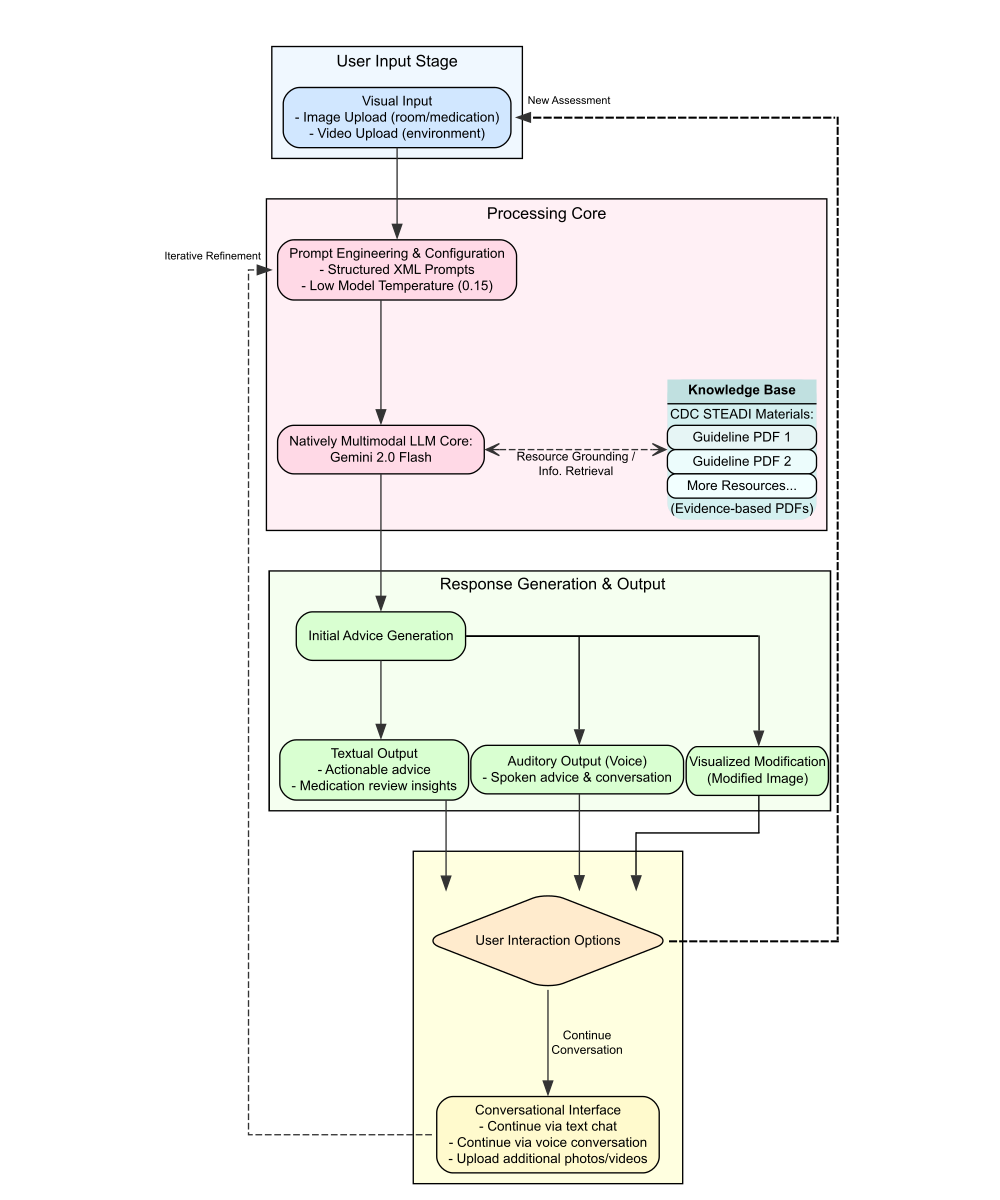
Enhanced model architecture for advice generation and multimodal interaction. CDC: Centers for Disease Control and Prevention; LLM: large language model; STEADI: Stopping Elderly Accidents, Deaths, & Injuries.

We selected Google’s Gemini family due to its strong visual reasoning performance supported by validated benchmarks [8]. We designed our framework to focus on providing reliable output by employing a low model temperature (0.15), in-context learning through grounding responses in evidence-based CDC STEADI patient materials (Figure 1), and structured XML prompts iterated using artificial intelligence (AI)-driven prompt engineering. The core innovation of this study used the gemini-2.0-flash-exp-image-generation model to directly modify the uploaded images with the model’s suggested changes. The model leverages a two-shot prompting system (Multimedia Appendix 1, Multimedia Appendix 2), in which the primary LLM generates textual recommendations based on an input image/video, then directs the image generation model to visually render these changes (eg, adding grab bars, removing hazards) onto the original image, iterating until the generated image reflects the proposed modifications. We conducted a formative, blinded, paired comparison of outputs generated from 27 publicly licensed “lived-in” home interior images. We compared a non-optimized baseline prompt with an enhanced multimodal pipeline (“Steadi”). Text and image outputs were compared based on clinical usefulness, safety, image fidelity/plausibility, and preference between baseline prompt output and our enhanced multimodal pipeline output. Detailed methods and output can be found in MultimediaAppendices 3  4.

## Results

### Initial Advice Generation, Multimodal Communication, and Modification Visualization

The model takes an uploaded image or video and provides specific, actionable advice supported by evidence-based resources. The proposed architecture successfully applies both additive and subtractive modifications to images, providing users with a concrete visual representation of a safer environment.

### Model Comparisons

We find that overall, our raters preferred the “Steadi” system output of both image and text (40/54 times (74.1%), Table 1). We demonstrate that contemporary LLMs produce relatively safe recommendations, regardless of the prompting system, with only one set of recommendations rated as unsafe in the baseline prompting system and none in the enhanced. We find that when given specific issues to visualize, image editing LLMs produce edits with good visualization fidelity (46/54 times, 85.2% for baseline and 43/54 times, 79.6% for enhanced; Table 1), and low rates of implausible/hazard-producing edits (6/54 times, 11.1% for both systems; Table 1). Text recommendations and visualized outputs ranged from generally “somewhat actionable” for the baseline system to highly actionable for our enhanced system.

**Table 1.** Results of rating study.

Outcome	Baseline	Enhanced	Comparison^a^
Q1 Overall clinical usefulness (preference)	Preferred: 10/54 (18.5%)	Preferred: 40/54 (74.1%)	Win rate: 40/50 (80.0%, 95% CI 67.0‐88.8); sign test *P*≤.001; ties=4
Q2 Unsafe/inappropriate recommendation rate (Yes)	1/54 (1.9%, 95% CI 0.3‐9.8)	0/54 (0.0%, 95% CI 0.0‐6.6)	Risk difference (Enhanced-Baseline): −1.9 pp
Q3 Visualization fidelity rate (Yes)	46/54 (85.2%, 95% CI 73.4‐92.3)	43/54 (79.6%, 95% CI 67.1‐88.2)	Risk difference (Enhanced-Baseline): −5.6 pp
Q4 Hazard-introducing/implausible edit rate (Yes)	6/54 (11.1%, 95% CI 5.2‐22.2)	6/54 (11.1%, 95% CI 5.2‐22.2)	Risk difference (Enhanced-Baseline):+0.0 pp
Q5 Actionability (1‐5 Likert)	median 3.0 (IQR 3.0‐4.0)	median 5.0 (IQR 4.0‐5.0)	Δmedian (Enhanced-Baseline):+1.0 (IQR 0.0‐2.0)

a n=rater case evaluations; outcomes are descriptive; Q1 sign test is exploratory.

## Discussion

### Principal Findings

This study introduces a novel application of multimodal LLMs, leveraging their image-generation capabilities for visualizing personalized home safety recommendations. We demonstrate that enhanced frameworks, such as structured prompting and grounding using trusted resources, produce safe, clinically useful, and actionable outputs that categorically rate better than outputs from baseline LLMs. The inherent flexibility of LLMs supports diverse interaction methods, uniquely enabling users to interact with their “consultant” in their preferred mode. LLMs may mitigate delays caused by insurance authorizations and restore autonomy to users.

The visual output capability is also key: generating suggestions directly onto uploaded images offers more intuitive, actionable guidance than abstract text instructions alone. The drive to protect the familiarity of their home from change was identified to be a major motive for older adults rejecting modification advice from OT [5]; direct visualization of user-fed images may help overcome this hurdle and increase acceptance. There are still limitations to the technology, namely outputs may be illogical such as the recommended soap placement, and movement of furniture and door in Multimedia Appendix 1. However, overall, this study demonstrates that LLMs generally produce visual outputs with high fidelity, low hazard introduction rates, and high actionability.

To adhere to HIPAA (Health Insurance Portability and Accountability Act) compliance, future work should consider working with LLM providers to sign a HIPAA Business Associate Amendment or other HIPAA-compliant program. Ethical considerations, such as disclosure of privacy and data protection, should be implemented in accordance with WHO guidance on AI in health [9].

### Limitations

Further testing must be conducted against the current standard for in-home assessments to discover if the proposed model provides comparable advice to professionals. Implementation trials will be needed to mitigate concerns such as the digital divide and ensure accessibility among varying cognitive/visual functions. Implementations must comply with FDA digital health guidance, and characterization and limitation of unsafe output generation must be explored. This model is designed as a supplemental service to be integrated with OT rather than a replacement.

### Conclusions

Multimodal LLMs that integrate image generation offer a novel, innovative approach to increasing end users’ accessibility to personalized home environment recommendations for fall prevention. This capability represents a potential supplement to current care services that may enhance patient understanding, motivation, and adherence, serving as a valuable resource to patients who defer or cannot access in-home safety assessments. Rigorous validation of clinical efficacy and user acceptance is essential to translate this technological potential into improved patient outcomes.

## Supplementary material

10.2196/77591 Multimedia Appendix 1 Two-shot image modification architecture.

10.2196/77591 Multimedia Appendix 2 XML Prompt and model parameters.

10.2196/77591 Multimedia Appendix 3 Blinded comparison methods.

10.2196/77591 Multimedia Appendix 4 Rating packet.

10.2196/77591 Multimedia Appendix 5 Usage of model for recommendations for post-stroke patients.

## Abbreviations

AI: artificial intelligence
CDC: Centers for Disease Control and Prevention
HIPAA: Health Insurance Portability and Accountability Act
LLM: large language model
MMMU: Massive Multi-discipline Multimodal Understanding and Reasoning
NICE: National Institute for Health and Care Excellence
OT: occupational therapy
STEADI: Stopping Elderly Accidents, Deaths, & Injuries
XML: Extensible Markup Language
