# Supplementary figures and images for: Leveraging Multimodal Large Language Models for Fall Risk Reduction in Older Adults in the Home: Proposed Model Design
Source: JMIR Aging. 2026 May 13;9:e77591. doi: 10.2196/77591 (PMC13170740; doi:10.2196/77591)

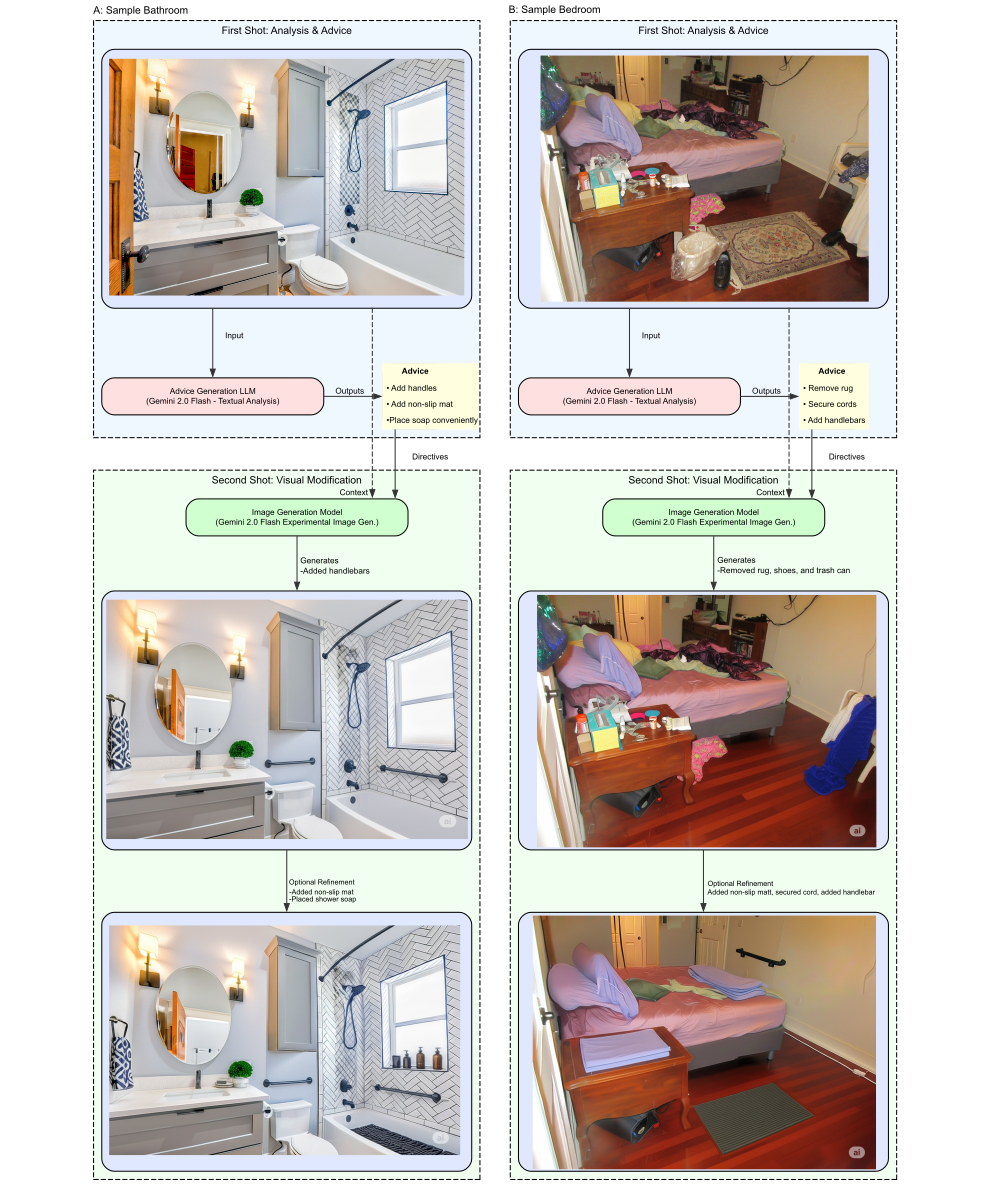

Supplement: Multimedia Appendix 1 [file aging-v9-e77591-s001.png]

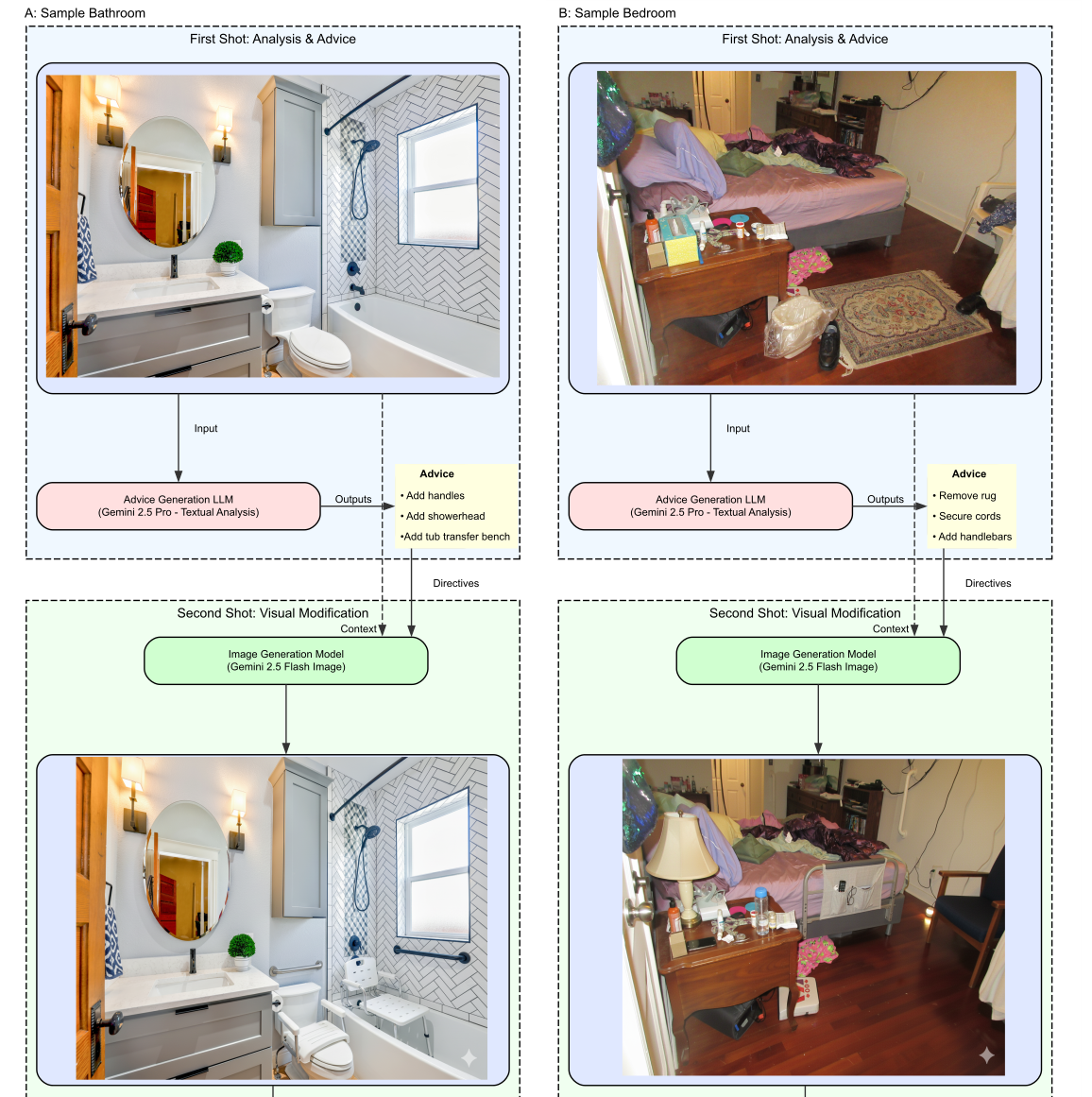

Supplement: Multimedia Appendix 5 [file aging-v9-e77591-s005.png]
