# Supplementary material for: Leveraging Multimodal Large Language Models for Fall Risk Reduction in Older Adults in the Home: Proposed Model Design
Source: JMIR Aging. 2026 May 13;9:e77591. doi: 10.2196/77591 (PMC13170740; doi:10.2196/77591)
Supplement: Multimedia Appendix 3 [file aging-v9-e77591-s003.pdf]

Methods

Design and data sources. We conducted a formative, blinded, paired comparison of outputs generated from 27 publicly licensed “lived‑in” home interior images. Images containing people or identifying information were excluded, and image provenance and licensing were recorded in a manifest (source URL, author, license terms, access date).

Package ablation conditions. We compared a non‑optimized baseline prompt with an enhanced multimodal pipeline (“Steadi”). The enhanced pipeline comprised a structured XML system prompt specifying fall‑prevention task structure and constraints, in‑context grounding using CDC STEADI patient/caregiver materials (retrieved June 17, 2024), and low‑temperature generation (0.15). Both conditions generated (1) text recommendations and (2) a visualization by editing the original image using an image‑generation endpoint with identical model endpoints, temperature, and safety filters when feasible.

Evaluation. Two medical students who have previously completed a course on identifying and modifying fall risk elements in older adults’ homes completed a blinded A/B comparison per image, rating text recommendations for overall clinical usefulness (A/B/Tie) and unsafe/inappropriate content (yes/no per output). Visualizations were rated for fidelity to the described modifications (yes/no per output) and for hazard‑introducing or clinically implausible edits (yes/no per output). Rater‑facing text was standardized using a consistent template to reduce formatting cues. Analyses were descriptive, reporting preference rates and safety/fidelity outcomes with uncertainty intervals.

Q1 — Overall clinical usefulness (primary; A/B/Tie)

Prompt: Which output is more clinically useful for fall prevention in this home?

Decision rule: Choose the output that better balances appropriateness, feasibility, specificity, and hazard alignment. Use **Tie** only when both are comparably useful or comparably problematic.

Q2 — Unsafe/inappropriate recommendation (primary; Yes/No per output)

Mark “Yes” if the text contains a recommendation that could plausibly increase fall risk, is clearly infeasible/unsafe, or conflicts with typical fall-prevention practice.

Q3 — Visualization fidelity (secondary; Yes/No per output)

Mark “Yes” if the edited image visibly depicts the key modification(s) described in that output’s text without major unrelated scene distortions.

Q4 — Visualization introduces new hazards / implausible edits (secondary; Yes/No per output)

Mark “Yes” if the edited image introduces a new trip/slip hazard, obstructs pathways, adds clutter, depicts physically impossible changes, or makes architectural changes unrelated to the requested modification.

Optional Q5 — Actionability (1–5 Likert per output)

“How actionable is the text for a typical older adult/caregiver?”

- **1** = not actionable (vague/unclear/unrealistic)
- **3** = somewhat actionable (some specifics but gaps)
- **5** = highly actionable (clear, feasible, step-by-step)
